# Bioelectric patterning during oogenesis: stage-specific distribution of membrane potentials, intracellular pH and ion-transport mechanisms in *Drosophila* ovarian follicles

**DOI:** 10.1186/s12861-015-0051-3

**Published:** 2015-01-16

**Authors:** Julia Krüger, Johannes Bohrmann

**Affiliations:** RWTH Aachen University, Institut für Biologie II, Abt. Zoologie und Humanbiologie, Worringerweg 3, 52056 Aachen, Germany

**Keywords:** *Drosophila melanogaster*, Oogenesis, Bioelectricity, Cell communication, Pattern formation, Membrane potential, Ion channel, Ion pump, Gap junction, Live-cell imaging

## Abstract

**Background:**

Bioelectric phenomena have been found to exert influence on various developmental and regenerative processes. Little is known about their possible functions and the cellular mechanisms by which they might act during *Drosophila* oogenesis. In developing follicles, characteristic extracellular current patterns and membrane-potential changes in oocyte and nurse cells have been observed that partly depend on the exchange of protons, potassium ions and sodium ions. These bioelectric properties have been supposed to be related to various processes during oogenesis, e. g. pH-regulation, osmoregulation, cell communication, cell migration, cell proliferation, cell death, vitellogenesis and follicle growth. Analysing in detail the spatial distribution and activity of the relevant ion-transport mechanisms is expected to elucidate the roles that bioelectric phenomena play during oogenesis.

**Results:**

To obtain an overview of bioelectric patterning along the longitudinal and transversal axes of the developing follicle, the spatial distributions of membrane potentials (V_mem_), intracellular pH (pH_i_) and various membrane-channel proteins were studied systematically using fluorescent indicators, fluorescent inhibitors and antisera. During mid-vitellogenic stages 9 to 10B, characteristic, stage-specific V_mem_-patterns in the follicle-cell epithelium as well as anteroposterior pH_i_-gradients in follicle cells and nurse cells were observed. Corresponding distribution patterns of proton pumps (V-ATPases), voltage-dependent L-type Ca^2+^-channels, amiloride-sensitive Na^+^-channels and Na^+^,H^+^-exchangers (NHE) and gap-junction proteins (innexin 3) were detected. In particular, six morphologically distinguishable follicle-cell types are characterized on the bioelectric level by differences concerning V_mem_ and pH_i_ as well as specific compositions of ion channels and carriers. Striking similarities between V_mem_-patterns and activity patterns of voltage-dependent Ca^2+^-channels were found, suggesting a mechanism for transducing bioelectric signals into cellular responses. Moreover, gradients of electrical potential and pH were observed within single cells.

**Conclusions:**

Our data suggest that spatial patterning of V_mem_, pH_i_ and specific membrane-channel proteins results in bioelectric signals that are supposed to play important roles during oogenesis, e. g. by influencing spatial coordinates, regulating migration processes or modifying the cytoskeletal organization. Characteristic stage-specific changes of bioelectric activity in specialized cell types are correlated with various developmental processes.

## Background

Oogenesis of *Drosophila melanogaster* provides an excellent model system for studying various aspects of cell and developmental biology, including cell migration, organization of the cytoskeleton, cell and tissue polarization, intra- and intercellular transport processes, signal transduction and determination of spatial coordinates. In several developing and regenerating systems, such cellular processes have been found to be regulated by bioelectric signals like membrane-potential gradients, ionic current patterns or domains of differing intracellular pH [1-4]. In *Drosophila* ovarian follicles, stage-specific extracellular current patterns [5,6] as well as membrane-potential changes in germ-line cells have been desribed that partly depend on the exchange of protons, potassium ions and sodium ions [7-9]. However, the possible functions of these bioelectric phenomena as well as the cellular mechanisms by which they might act have largely remained elusive.

*Drosophila* follicles consist of 16 germ-line cells - 15 nurse cells (NC) and one oocyte (Ooc) - that are surrounded by a layer of somatic follicle cells (FC). The germ-line cells form a cytoplasmic continuum, since they are connected via intercellular bridges (ring canals) as well as via gap junctions [10]. The same holds true for the somatic cells [11], whereas germ-line and somatic cells are interconnected only via gap junctions [12]. Besides these communication paths, a variety of intercellular signaling mechanisms play a role during follicle development, since the formation of a mature egg, surrounded by a functional eggshell and containing information for embryonic axes, requires a wealth of specifications as well as patterning and symmetry-breaking steps [13].

**Figure 1:**
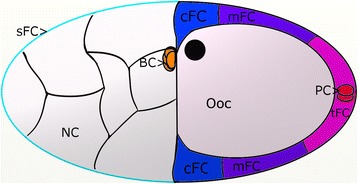
**Six follicle-cell types are distinguishable on the morphological and on the molecular level.** Schematic drawing, S10B. Each population is indicated by a different colour and consists of multiple cells which are not shown in detail, except for the polar cells (2 cells) and the border cells (6–10 cells). Anterior is to the left. The position of the oocyte nucleus (black) marks the dorsal side. BC: border cells, cFC: centripetal follicle cells, mFC: mainbody follicle cells, NC: nurse cells, Ooc: oocyte, PC: polar cells, sFC: stretched follicle cells, tFC: terminal follicle cells.

According to morphological criteria, *Drosophila* oogenesis has been divided into 14 stages (S1 to S14). In the FC epithelium, 6 types of cells can be distinguished on the morphological as well as on the molecular level during mid-vitellogenic stages 9 to 10B [14-16] (Figure 1). The terminal FC (tFC) are a population of about 200 cells at the posterior follicle pole. The mainbody FC (mFC) form a broad band anterior to the tFC. The centripetal FC (cFC) reside at the anterior end of the Ooc and migrate in between NC and Ooc during S10B. The stretched FC (sFC) become flattened and cover the NC. A pair of polar cells (PC) resides at the posterior pole of the Ooc. The border cells (BC), a group of 6–10 anterior FC, migrate between the NC from the anterior follicle pole to the anterior pole of the Ooc during S9.

In order to investigate bioelectric phenomena in more detail, we compared membrane potentials (V_mem_) and intracellular pH-values (pH_i_) of different cell types using a potentiometric probe and a fluorescent pH-indicator. In addition, we localized various membrane-channel proteins, namely vacuolar-type H^+^-ATPases (V-ATPases), voltage-dependent L-type Ca^2+^-channels, amiloride-sensitive Na^+^-channels and Na^+^,H^+^-exchangers as well as the gap-junction protein innexin 3.

The spatial distribution of V-ATPases was analysed immunohistochemically in order to detect interrelations between V-ATPase activity and the pH_i_- and V_mem_-patterns, especially in different FC types. V-ATPases are a family of proton pumps composed of multiple subunits [17,18], that are required for the acidification of intracellular compartments [19,20] and are located in the plasma membranes of many epithelia. Active proton translocation by V-ATPases is electrogenic and has influences on intra- and extracellular pH. The proton gradient is used to drive secondary active antiport via different mechanisms, for example Na^+^,H^+^-exchangers (NHE), Na^+^,K^+^,Cl^−^-cotransporters and K^+^,H^+^-exchangers [21-23]. For ovarian follicles of several insects, including *Drosophila,* a role of V-ATPases in energizing the plasma membranes has been proposed [24-26]. Moreover, V-ATPases have been found to influence various developmental processes, like cell division, cell migration and differentiation [27,28]. For example, in vertebrate embryos, the activity of V-ATPases and its resulting pH_i_- and V_mem_-patterns play a role during the formation of left-right asymmetries [29].

One possible mechanism for the transduction of bioelectric signals, like V_mem_-alterations, into cellular responses is Ca^2+^-flux through voltage-dependent channels [30]. We, therefore, analysed the spatial distribution of L-type Ca^2+^-channels using a specific fluorescent inhibitor as well as immunohistochemistry. Members of this channel family are sensitive to V_mem_-changes and are characterized through a pharmacological sensitivity to 1,4-dihydropyridines and phenylalkylamines mediated by the α1-subunit of the protein [31,32]. L-type Ca^2+^-channels appear in three functionally distinct states: resting, or closed, channels, activated, or open, channels, and inactivated channels. In nerve cells, depolarization results in a conformational change from the resting to the activated state and then to rapid inactivation, while repolarization is necessary to return to the resting state [32]. The binding affinity of phenylalkylamines, like verapamil, depends on the channel’s state in the following order: inactivated > > activated > resting [33,34]. Voltage-dependent L-type Ca^2+^-channels were originally considered to be unique to excitable cells [35], but now they are known to be present in non-excitable cells as well. For example, they play a role in Ca^2+^-reabsorption in mammalian renal epithelia [36] and in epithelial fluid transport in *Drosophila* Malpighian tubules [37].

The spatial distribution of amiloride-sensitive NHE and Na^+^-channels was investigated using fluorescent amiloride. NHE are important for pH_i_-homeostasis, cell-volume regulation and transepithelial Na^+^-transport [38]. In insect epithelia, the electroneutral exchange of Na^+^ against H^+^ through NHE is driven by the H^+^-gradient generated by V-ATPases [21,39]. Amiloride-sensitive Na^+^-channels have been found to play a role during reproductive as well as developmental processes. For example, they help to block polyspermy in *Xenopus* oocytes [40] and, in *Drosophila*, the amiloride-sensitive Na^+^-channel *dGNaC1* is expressed in gonads and early embryos. This channel has been proposed to be involved in cytoplasmic transport processes and in water uptake during final maturation of the Ooc [41].

Members of the innexin family are the main gap-junction proteins in invertebrates [42,43], although other proteins, like pannexins [44] and ductins [45-48], have also been observed in gap junctions. In the *Drosophila* ovary, the mRNAs of innexins 1, 2, 3, 4 and 7 were detected [49] and innexins 1 to 4 have been shown immunohistochemically to be involved in the formation of different types of gap junctions [50]. Since innexins 1, 2 and 4 did not show conspicuous spatial distribution patterns, we only present the distribution of innexin 3 in more detail.

Using fluorescent indicators, inhibitors and antisera, we found corresponding asymmetries in the distribution patterns along the longitudinal and transversal axes of the follicle during S9 to S10B. Characteristic stage-specific changes observed in different cell types are correlated with various cellular and developmental processes.

## Methods

### Preparation of follicles

*Drosophila melanogaster* wild-type Oregon R flies were reared at about 20°C on standard food with additional fresh yeast. Individual 2–3 days old females were killed by crushing the thorax with tweezers without previous etherization or chilling. The ovaries were dissected with tweezers, and single follicles of S9 to S10B were isolated by pulling at the anterior tip of an ovariole. For immunohistochemistry, dissection was carried out in *Drosophila* PBS [51], while for *in-vitro* experiments with fluorescent inhibitors and indicators R-14 medium [51] was used, which ensures optimal culture conditions during live-cell imaging of follicles [52].

### Antisera

To localize V-ATPases, we used two antisera: (1) a rabbit antiserum (Anti-ductin; AB5496, Chemicon International, USA) raised against a highly conserved region of the 16 kDa-protein ductin, which forms subunit c of V-ATPases and is also part of gap junctions [48], and (2), as a control, a guinea-pig antiserum (Anti-V-ATPase) raised against an N-terminal region of subunit a of the *Manduca sexta* V-ATPase, kindly provided by B. Walz and O. Baumann (Potsdam, Germany). For the localization of innexin 3, we used a guinea-pig antiserum (Anti-Inx3 [50]) raised against the C-terminus of innexin 3 from *Drosophila*, kindly provided by R. Bauer and M. Hoch (Bonn, Germany). In control experiments, L-type Ca^2+^-channels were localized using a rabbit antiserum (Anti-Ca_v_α1; Anti-Ca_v_panα1 subunit, Almone Labs, Israel) raised against an intracellular epitope of subunit α1.

### Indirect immunofluorescence preparations

Follicles were fixed for 30 minutes at 4°C in 4% formaldehyde dissolved in PBS, washed in PBS and blocked for 1 hour at 20°C with 2% BSA/0,1% Triton X-100 in PBS. Thereafter, the follicles were incubated overnight at 4°C in PBS containing 0,5% BSA/0,1% Triton X-100 and the respective antiserum (Anti-ductin diluted 1:100, Anti-V-ATPase diluted 1:1000, Anti-Inx3 diluted 1:20, Anti-Ca_v_α1 diluted 1:100, controls without antiserum).

After washing 6 times for 10 minutes, the follicles were either treated with goat-anti-rabbit-Cy3 (Jackson, USA; diluted 1:2000) or with donkey-anti-guinea-pig-FP488 (FluoProbes/Interchim, France; diluted 1:100) for 1 hour in PBS containing 0,5% BSA/0,1% Triton X-100. Washing was repeated 6 times and the nuclei were stained with 0,2 μg/ml DAPI (Sigma, Germany) in PBS for 3 minutes. Thereafter, the follicles were mounted in Fluoromount G (Interchim) and viewed, by using ×20 or ×40 objectives and the appropriate filter sets, either on a Zeiss Axiovert 200 wide-field fluorescence microscope (WFM), equipped with a Hamamatsu Orca ER camera, or on a Zeiss AxioImager.M2 structured-illumination microscope (SIM), equipped with a Zeiss ApoTome and a Zeiss AxioCamMRm camera.

### Fluorescent inhibitors

Staining of living follicles with fluorescent inhibitors was carried out in R-14 medium. Stock solutions were prepared in DMSO, and the follicles were incubated either in 2 μM amiloride-FL (BODIPY-FL amiloride; Molecular Probes/Thermo Fisher Scientific, USA) or in 2 μM verapamil-FL (BODIPY-FL verapamil, hydrochloride; Molecular Probes) for 15 minutes. Control follicles were preincubated before labeling with the unlabeled inhibitors (Sigma, Germany; stock solutions in ethanol) for 15 minutes using 10 μM amiloride or 100 μM verapamil-HCl (or they were labeled with Anti-Ca_v_α1). Thereafter, the follicles were mounted in R-14 medium and viewed immediately as described above.

### Fluorescent membrane-potential indicator

In order to analyse V_mem_-patterns, we used the fluorescent potentiometric probe DiBAC (bis-(1,3-dibutylbarbituric acid) trimethine oxonol, DiBAC_4_(3); Molecular Probes). The anionic dye enters cells and binds to intracellular membranes and proteins in a V_mem_-dependent manner: depolarization leads to an accumulation and to an increase in fluorescence intensity. Relative fluorescence differences between cells of comparable size were stated, i. e. stronger fluorescence: more depolarized vs. weaker fluorescence: more hyperpolarized. Living follicles were incubated for 15 minutes in R-14 medium containing 1–3 μM DiBAC (dissolved in 70% ethanol). Thereafter, they were mounted in R-14 medium and viewed immediately as described above.

### Fluorescent intracellular pH-indicator

For the analysis of pH_i_-patterns, we used the fluorescent pH-indicator CFDA (5-carboxyfluorescein diacetate, acetoxymethyl ester, 5-CFDA, AM; Molecular Probes). The anionic dye enters cells and reports pH_i_ by fluorescence-intensity differences, since protonation leads to fluorescence loss. Relative fluorescence differences between cells of comparable size were stated, i.e. stronger fluorescence: more alkaline vs. weaker fluorescence: more acidic. Living follicles were incubated for 15 minutes in R-14 medium containing 4 μM CFDA (dissolved in DMSO), and then mounted and viewed as described above.

### Analysis of staining patterns

To facilitate interpretation and comparability of the patterns obtained with different staining methods, we analysed median optical sections through the follicles. Representative grey-scale images were transferred into pseudocolour images using ImageJ Fire-LUT (NIH, USA). For immunostaining as well as fluorescent inhibitors, brighter colours represent higher concentrations of the respective membrane-channel protein. For DiBAC, brighter colours refer to more depolarized V_mem_, and for CFDA, brighter colours refer to more alkaline pH_i_. Each experiment was repeated at least three times.

## Results

### Gradients of membrane potentials in follicle cells and oocyte

**Figure 2:**
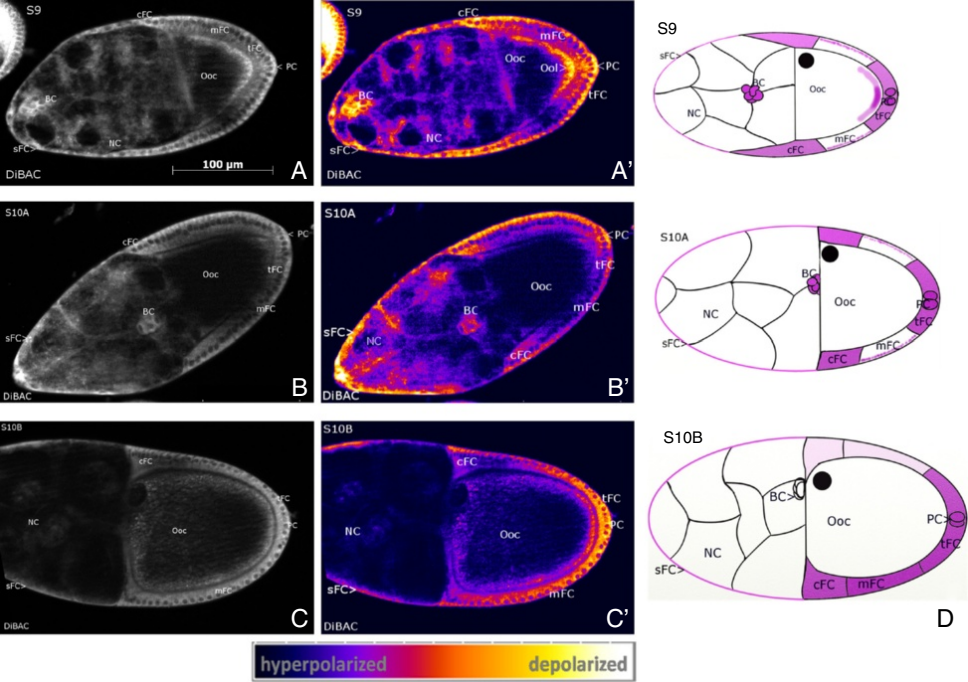
**Characteristic patterns of membrane potentials (V**_**mem**_ **).** DiBAC staining, SIM, S9 to S10B, **A, B, C**: representative grey-scale images, **A´, B´, C´:** corresponding pseudocolour images. Depolarization is indicated by strong, hyperpolarization by weak fluorescence intensity (see scale bar). **D:** schematic drawings of characteristic V_mem_-patterns; depolarized regions are shown in lilac (staining in NC is not considered). In S9 **(A, A´)** the FC epithelium is patterned along the anteroposterior axis. Strongest depolarization is found in cFC, sFC, tFC, BC and PC. The mFC are characterized by a hyperpolarized V_mem_ and an intracellular apicobasal gradient (apical region near Ooc is hyperpolarized compared to basal region), while the anterior region of the Ooc is hyperpolarized relative to the posterior region. In S10B **(C, C´)** the ventral side of the follicle is depolarized (arrow) in relation to the other side. S10A **(B, B´)** shows characteristics of both S9 and S10B. For abbreviations, see Figure 1.

Using the potentiometric probe DiBAC, we revealed stage specific V_mem_-patterns during S9 to S10B by comparing fluorescence intensities in different follicle regions. Stronger intensities refer to more depolarized V_mem_, weaker intensities to more hyperpolarized V_mem_. In S9 (Figure 2A) we found a complex V_mem_-pattern in the FC epithelium: mainbody FC (mFC) are characterized by a hyperpolarized V_mem_ in relation to the neighboring terminal FC (tFC, posterior), centripetal FC (cFC, anterior) and stretched FC (sFC, further anterior). In each mFC, an apicobasal gradient was observed, the apical region being more hyperpolarized than the basal region. The strongest depolarization was found in migrating border cells (BC) and in posterior polar cells (PC). In the oocyte (Ooc), an anteroposterior gradient was observed, the anterior region being more hyperpolarized than the posterior region. During S10B (and S11, data not shown) we found a transversal V_mem_-pattern, where one side of the FC epithelium (including tFC, mFC and cFC) was depolarized in relation to the opposite side. In most follicles, the depolarized side could be identified as the ventral side (25 vs. 3 follicles) according to the location of the Ooc nucleus (DIC-microscopy). S10A showed characteristics of both S9 and S10B: mFC are hyperpolarized in relation to cFC and tFC, but one side of the FC epithelium is already more depolarized than the other (Figure 2B and C).

### Gradients of intracellular pH in nurse cells and follicle cells

**Figure 3:**
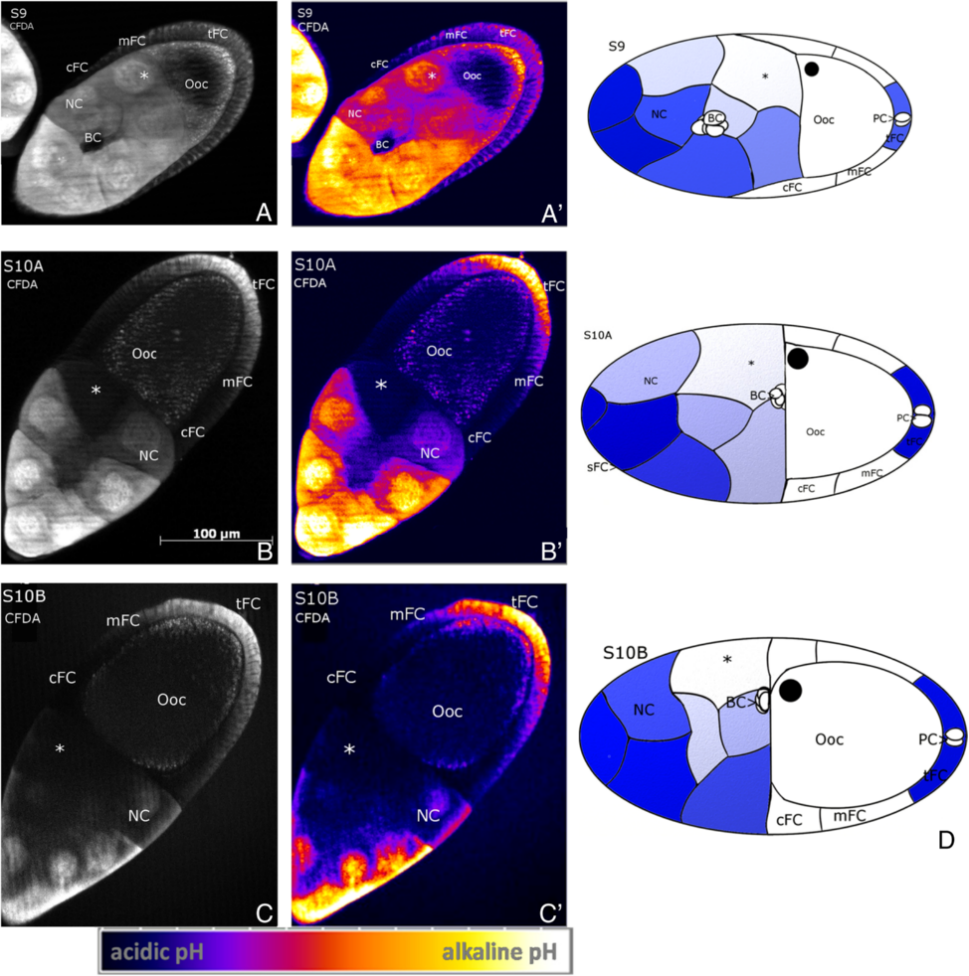
**Characteristic patterns of intracellular pH (pH**_**i**_ **).** CFDA staining, SIM, S9 to S10B, **A, B, C**: representative grey-scale images, **A´, B´, C´**: corresponding pseudocolour images. Alkaline pH_i_ is indicated by strong, acidic pH_i_ by weak fluorescence intensity (see scale bar). **D**: schematic drawings of characteristic pH_i_-patterns; alkaline regions are shown in blue (staining in Ooc is not considered). Between S9 and S10B, an anteroposterior pH_i_-gradient develops within the NC group, the NC adjacent to the anterodorsal region of the Ooc (asterisk) being most acidic. Usually, an anteroposterior pH_i_-gradient was observed within the FC epithelium, the cFC being most acidic. Also the migrating BC are characterized through an acidic pH_i_. Near the posterior oolemma (Ool), the Ooc is alkaline compared to the remaining regions. For abbreviations, see Figure 1.

Using the pH-indicator CFDA, we analysed pH_i_-patterns in the developing follicle. A more acidic pH_i_ is indicated by weaker, a more alkaline pH_i_ by stronger fluorescence intensity. During S9 to S10B, we found stage-specific pH_i_-patterns in the NC and in the FC (Figure 3). In the NC cluster, remarkable anteroposterior and dorsoventral gradients emerged, where each NC is characterized through its own pH_i_: the NC adjacent to the anterodorsal region of the Ooc (location of the nucleus) has the most acidic pH_i_, and the anterior-most NC has the most alkaline pH_i_. Both gradients develop in S9 and become fully established during S10. In the FC epithelium, we found greater variability: most examined follicles showed an anteroposterior pH_i_-gradient, the sFC, cFC and mFC being most acidic and the tFC being most alkaline. Some follicles, especially in S9, also showed a transversal pH_i_-gradient in the FC epithelium, with one side being more acidic than the other (data not shown). In addition, in S9 the migrating BC are characterized through acidic pH_i,_ and the posterior region of the Ooc is more alkaline than the anterior region (Figure 3A).

### Asymmetric V-ATPase distribution in the follicle-cell epithelium

**Figure 4:**
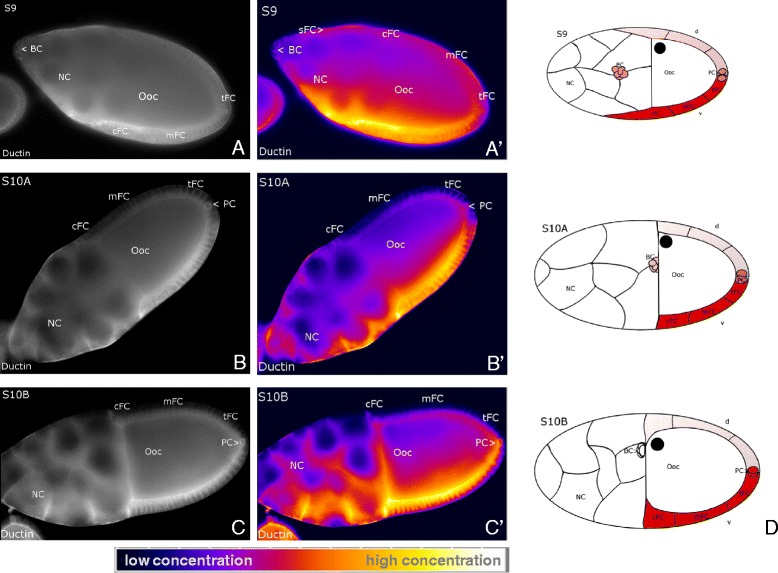
**Characteristic distribution patterns of V-ATPases.** Anti-ductin staining, WFM, S9 to S10B, **A, B, C**: representative grey-scale images, **A´, B´, C´**: corresponding pseudocolour images. V-ATPases are localized in lateral and apical FC membranes, in NC membranes and in the oolemma. Between S9 and S10B, an asymmetric distribution is found in the FC epithelium (see scale bar): V-ATPases are accumulated in the membranes of tFC, mFC, sFC and cFC on the ventral follicle side, and also in PC. In **D** the characteristic distribution patterns are summarized schematically; high concentrations of V-ATPases are shown in red (staining in Ooc and NC is not considered). For abbreviations, see Figure 1.

By indirect immunofluorescence microscopy using two different antisera (Anti-ductin and Anti-V-ATPase) we investigated the localization of V-ATPases during mid-vitellogenic stages. Since the 16 kDa-protein ductin is known to be part of both V-ATPases and gap junctions [48], we used Anti-V-ATPase as a control to decide, whether the observed distribution of ductin results from the distribution of V-ATPases or gap junctions. Both antisera recognized cytoplasmic as well as membranous antigens. Membrane labeling with Anti-ductin was either punctate or continuous (Figure 4), while with Anti-V-ATPase it was always continuous (data not shown). Punctate membrane labeling is presumed to originate from ductin as part of gap junctions, whereas continuous membrane labeling is presumed to originate from ductin as part of V-ATPases [26]. We found V-ATPases in lateral and in apical FC membranes, in NC membranes and in the oolemma, while in the FC epithelium, characteristic asymmetries in the spatial distribution were observed with both antisera (for Anti-V-ATPase, data not shown). Accordingly, V-ATPases (*not* ductin-containing gap junctions, which show uniform punctate distribution) are enriched in FC on one side of the follicle, including tFC, mFC, cFC and sFC, whereas FC on the opposite side contain considerably less. In most follicles, the enriched side could be identified as the ventral side (13 vs. 1 follicles) according to the location of the Ooc nucleus (DIC-microscopy). Also the membranes of PC showed accumulation of V-ATPases (Figure 4). Control follicles showed no specific staining (data not shown).

### L-type Ca^2+^-channel patterns correspond to membrane-potential patterns

**Figure 5:**
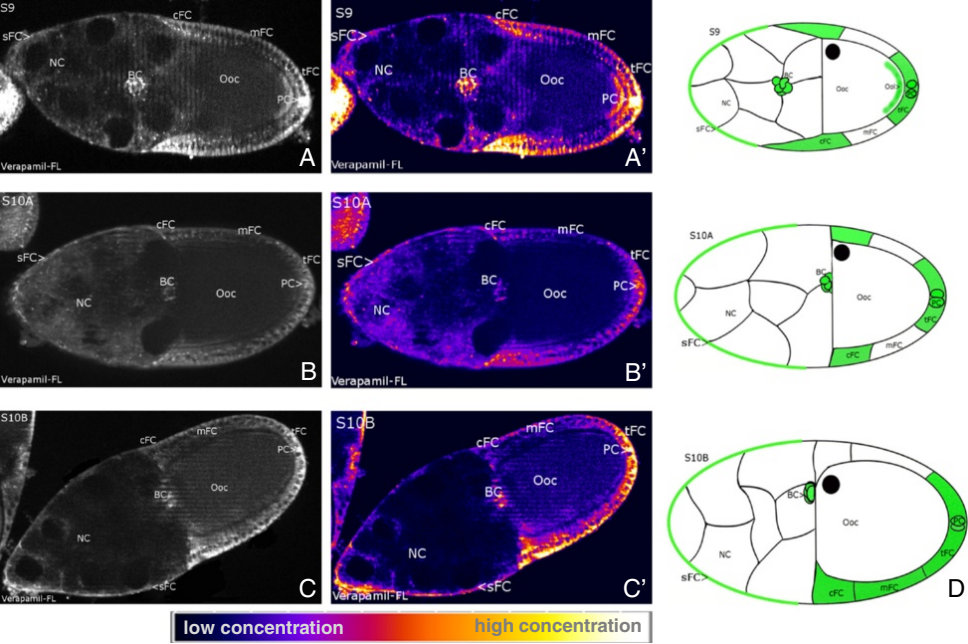
**Characteristic distribution patterns of voltage-dependent L-type Ca** ^**2+**^ **-channels.** Verapamil-FL staining, SIM, S9 to S10B, **A, B, C**: representative grey-scale images, **A´, B´, C´:** corresponding pseudocolour images. In S9 **(A, A´)** and S10A **(B, B´)** the concentration of labeled Ca^2+^-channels is considerably higher in tFC, cFC and sFC than in mFC (see scale bar). BC and PC show the highest concentrations of labeled Ca^2+^-channels in S9. In S10B **(C, C´)** the ventral side of the FC epithelium exhibits a considerably higher concentration of labeled, i. e. activated, or open, (and inactivated) channels than the dorsal side, where resting, or closed, channels predominate. In **D** the characteristic distribution patterns are summarized schematically; high concentrations of activated (and inactivated) Ca^2+^-channels are shown in green (staining in NC is not considered). For abbreviations, see Figure 1.

Using the fluorescent inihibitor verapamil-FL, we analysed the distribution of L-type Ca^2+^-channels. Since binding of the inhibitor depends on the channel’s conformational state, we used immunohistochemistry as a control to discriminate between distribution and activity patterns. While Anti-Ca_v_α1 revealed uniform distributions of L-type Ca^2+^-channels along the longitudinal and transversal follicle axes during mid-vitellogenic stages (data not shown), verapamil-FL showed asymmetric staining patterns (Figure 5), that are supposed to represent differences in the conformational state of these voltage-dependent channels. Verapamil binds with higher affinity to channels being exposed to depolarized V_mem_, which means that they are activated, or open (and inactivated). The distribution patterns of verapamil-FL-labeled channels were very similar to the V_mem_-patterns described above (Figure 2). Regions having more depolarized V_mem_ contained higher concentrations of supposed activated (and inactivated) channels. In S9 and S10A, these regions are the tFC, cFC and sFC as well as the BC and PC, while in the mFC the concentration of supposed resting, or closed, channels was higher.

In each mFC, verapamil-FL revealed an apicobasal gradient of activated (and inactivated) Ca^2+^-channels, with higher concentration on the depolarized basal side (Figure 5A and B). In S9 we found a higher concentration of such channels also in the posterior region of the Ooc, which is depolarized compared to the anterior region (Figure 2A). In S10B (Figure 5C) an asymmetric distribution along the transversal follicle axis was observed where one side contained a higher concentration of activated (and inactivated) Ca^2+^-channels than the other side. In control follicles preincubated with unlabeled verapamil the gradual staining disappeared (data not shown).

### Innexin-3-containing gap junctions are enriched in centripetal follicle cells

**Figure 6:**
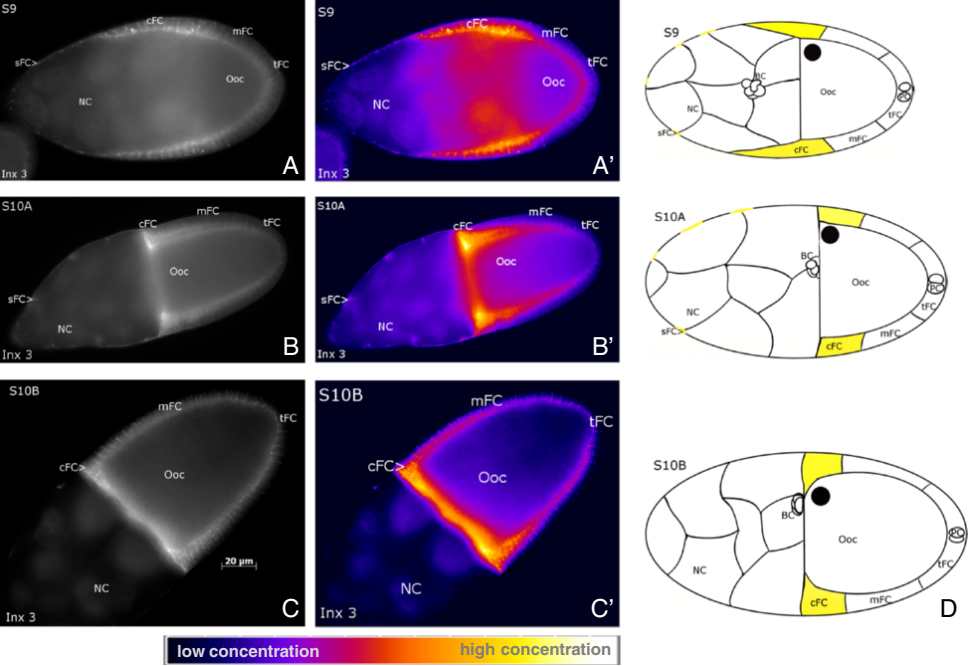
**Characteristic distribution patterns of innexin-3-containing gap junctions.** Anti-Inx3 staining, WFM, S9 to S10B, **A, B, C**: representative grey-scale images, **A´, B´, C´**: corresponding pseudocolour images. Innexin 3 is predominantly localized in lateral FC membranes, the highest concentration being found in cFC (see scale bar). In **D** the characteristic distribution patterns are summarized schematically; high concentrations of innexin 3 are shown in yellow (staining in NC is not considered). For abbreviations, see Figure 1.

Using immunohistochemistry, we have previousley analysed in ovarian follicles the localisation of the gap-junction proteins innexin 1 to 4 [50]. In the present study, we show that innexin 3 has a non-uniform distribution along the longitudinal follicle axis between S9 and S10B. As described earlier, Anti-Inx3 recognizes antigens in NC membranes and, especially, in lateral FC membranes, showing either continuous or punctate labeling [50]. Interestingly, innexin 3 is present in considerably higher amounts in the lateral membranes of cFC than of the other FC (Figure 6). Thus, cFC are especially interconnected via innexin-3-containing gap junctions (in addition to other innexin- and ductin-containing gap junctions [46,50]). Control follicles showed no specific staining (data not shown).

### Gradients of amiloride-sensitive Na^+^-channels and NHE in nurse cells

**Figure 7:**
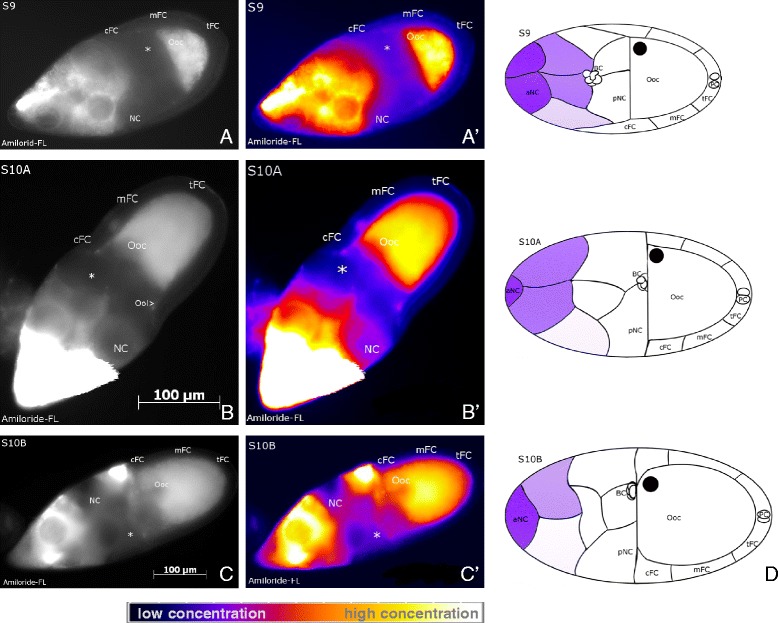
**Characteristic distribution patterns of Na** ^**+**^ **-channels and Na** ^**+**^ **,H** ^**+**^ **-exchangers.** Amiloride-FL staining, WFM, S9 to S10B, **A, B, C**: representative grey-scale images, **A´, B´, C´**: corresponding pseudocolour images. Amiloride-sensitive Na^+^-transporters are distributed in an anteroposterior gradient within the NC group. Usually, high concentrations were found in anterior NC with declining concentrations in posterior direction (see scale bar), the NC adjacent to the anterodorsal region of the Ooc (asterisk) having the lowest concentation. In **D** the characteristic distribution patterns are summarized, high concentrations of Na^+^-transporters are shown in purple (staining in Ooc is not considered). For abbreviations, see Figure 1.

To investigate the distributions of Na^+^-channels and Na^+^,H^+^-exchangers (NHE), we used the fluorescent inhibitor amiloride-FL. Since the inhibitor binds to both ion-transport mechanisms with high affinity, they cannot be discriminated with this method. Amiloride-FL mainly recognized these ion transporters in a non-uniform pattern in the NC cytoplasm (Figure 7), and, due to this intense cytoplasmic labeling, it was difficult to detect membrane labeling. During S9 to S10B, most follicles showed an anteroposterior gradient of NHE and Na^+^-channels, with the highest concentration in the anterior-most NC. Control follicles preincubated with unlabeled amiloride showed no specific staining (data not shown).

## Discussion

The activities of ion-transport mechanisms regulate intracellular ion composition, pH_i_ and V_mem_. Coordinated interactions of different ion channels, ion pumps and gap junctions result in bioelectric phenomena, like pH-gradients, voltage gradients and ion fluxes within single cells or tissues. It is known that bioelectricity can act as regulator in various developmental and regenerative processes, e. g. proliferation, differentiation, polarisation and migration [1,3].

The composition and activity of ion-transport mechanisms may vary in space and time leading to stage-specific changes in current flow and voltage gradients. Therefore, we analysed, during the course of *Drosophila* oogenesis, the spatial distributions of different membrane channels, V_mem_ and pH_i_. Especially during mid-vitellogenesis (S9 to S10B), we found characteristic stage-specific distributions that are supposed to provide bioelectric signals for epithelial patterning and morphogenesis.

### Ovarian follicles show characteristic patterns of both bioelectric activity and ion-transport mechanisms

Many aspects of patterning, axis formation and morphogenesis have been intensely studied in *Drosophila* ovarian follicles. Oogenesis involves hierarchically organized symmetry-breaking events [53], diverse reorganizations of the cytoskeleton [54,55], directed localization of cytoplasmic determinants [56], determination of cell fates and coordinated cell migrations [14-16]. All of these processes are mediated by successive interactions of signaling mechanisms, including cell contacts by cadherines and gap junctions, secreted signals, like EGF- and BMP-related proteins, and Notch signaling [57].

During S9 to S10B, we found striking stage-specific V_mem_- and pH_i_-patterns that correspond to populations of morphologically distinguishable FC types. In addition, a prominent anteroposterior pH_i_-gradient was observed within the NC cluster. We also revealed, along the longitudinal and/or transversal follicle axes, asymmetric distributions of membrane channels, namely V-ATPases, L-type Ca^2+^-channels, amiloride-sensitive Na^+^-channels and NHE, and innexin-3-containing gap junctions, that all are supposed to be involved in the regulation of V_mem_ and/or pH_i_. Actually, we found remarkable accordance between the stage-specific distributions of these membrane channels and the V_mem_- and pH_i_-patterns.

S9 and S10A are characterized through longitudinal bioelectric patterns, where the mFC are hyperpolarized in relation to the tFC, cFC and sFC. While the PC and the migrating BC are more depolarized, the BC are more acidic, and the tFC are more alkaline than the other FC. In addition, we found bioelectric gradients in the Ooc, with the posterior region being depolarized and alkaline in relation to the anterior region. S10B, however, is characterized through a transversal V_mem_-pattern, with the ventral side of the FC epithelium being more depolarized than the dorsal side.

### Voltage-dependent Ca^2+^-channels are candidates for transducing V_mem_-alterations into cellular responses

Close accordance was observed between the patterns of V_mem_ and the activity patterns of voltage-dependent L-type Ca^2+^-channels. All regions being more depolarized exhibited intense verapamil-FL fluorescence, indicating high concentrations of channels in the activated, or open (and inactivated) state, which depends on a depolarized V_mem_. This finding points to a mechanism by which alterations of V_mem_ could be transduced into cellular responses through voltage-dependent Ca^2+^-currents. Equally distributed Ca^2+^-channels (indicated by Anti-Ca_v_α1) are supposed to respond to stage-specific regional changes of V_mem_ and, thereby, regulate intracellular Ca^2+^-concentrations in discrete follicle regions. Ca^2+^-ions are known to act as signaling molecules in many developing systems e.g. [58,59]. Ca^2+^-signaling in the FC epithelium of *Drosophila* has not been addressed so far, but influences on the cytoskeleton, e.g. on contractions of the basal actomyosin network [60], seem to be likely.

### V-ATPases are candidates for generating V_mem_-patterns

Using antisera raised against different components of V-ATPases, we revealed an asymmetric distribution of proton pumps in the FC epithelium. During S9 to S10B, V-ATPases are accumulated in the lateral and apical plasma membranes on the ventral side of the follicle, including tFC, mFC, cFC and sFC. Since the activity of V-ATPases in epithelia is electrogenic, the pumps are supposed to have direct or indirect influence on V_mem_ in ovarian follicles. This hypothesis is supported by the accordance between the dorsoventral V-ATPase-pattern during S9 to S10B and the dorsoventral V_mem_-pattern in S10B. Since, besides their primary functions, V-ATPases are also known to play specialized roles in developing systems e.g. [29], their accumulation on one follicle side points to a possible role during the regulation of the spatial coordinates.

### Na^+^-transporter distribution correlates with a pH_i_-gradient in nurse cells

Amiloride-sensitive Na^+^-channels and NHE showed high concentrations in anterior and low concentrations in posterior NC. This gradient corresponds to a pH_i_-gradient with a most alkaline anterior NC and a most acidic posterior NC adjacent to the anterodorsal region of the Ooc. Therefore, an interrelation between amiloride-sensitive Na^+^-transport and pH_i_ is very likely. Either Na^+^-transport, probably through NHE in exchange to H^+^, is involved in the establishment of the pH_i_-gradient, or the pH_i_-gradient exerts influence on the activity of Na^+^-transporters. Despite cytoplasmic coupling via ring canals and gap junctions, each NC regulates its pH_i_ independently. The resulting anteroposterior pH_i_-gradient is supposed to play a role in various processes during oogenesis. In general, pH_i_ is known to exert influence on cell metabolism, enzyme activity, contractibility of cytoskeletal structures, cell polarity and proliferation rates of cells [61-63]. During S9, the pH_i_-gradient might also provide guidance cues for BC migration.

### Communication via specialized gap junctions is likely to generate and maintain boundaries between different cell populations

Of the ovarian gap-junction proteins innexin 1 to 4, only innexin 3 showed a striking non-uniform distribution within the FC epithelium: it is enriched in the lateral membranes of cFCs. This finding indicates distinct coupling conditions between these cells, concerning e.g. regulatory signals, V_mem_ and pH_i_. Moreover, innexin 3 seems to be involved in maintaining tissue integrity in response to tension [64], a function that is particularly important for cFC.

The cFC are specialized in several further respects: they are longer dye-coupled to the Ooc by gap junctions [12], they contain higher amounts of Na^+^,K^+^-ATPase [26,65] (which becomes activated under alkaline conditions [66]), and they possess a higher [Ca^2+^]_i_ than the remaining FC [67]. The permeability of innexin channels in general has been reported to be V_mem_-, pH_i_-, K^+^- and Ca^2+^-sensitive [68,69]. Restriction to distinct cell populations and independent regulation of permeability of specialized gap-junction channels (formed by different proteins in either homomeric, heteromeric or heterotypic combinations [50]) could provide mechanisms to generate or maintain boundaries between different FC populations.

## Conclusions

**Figure 8:**
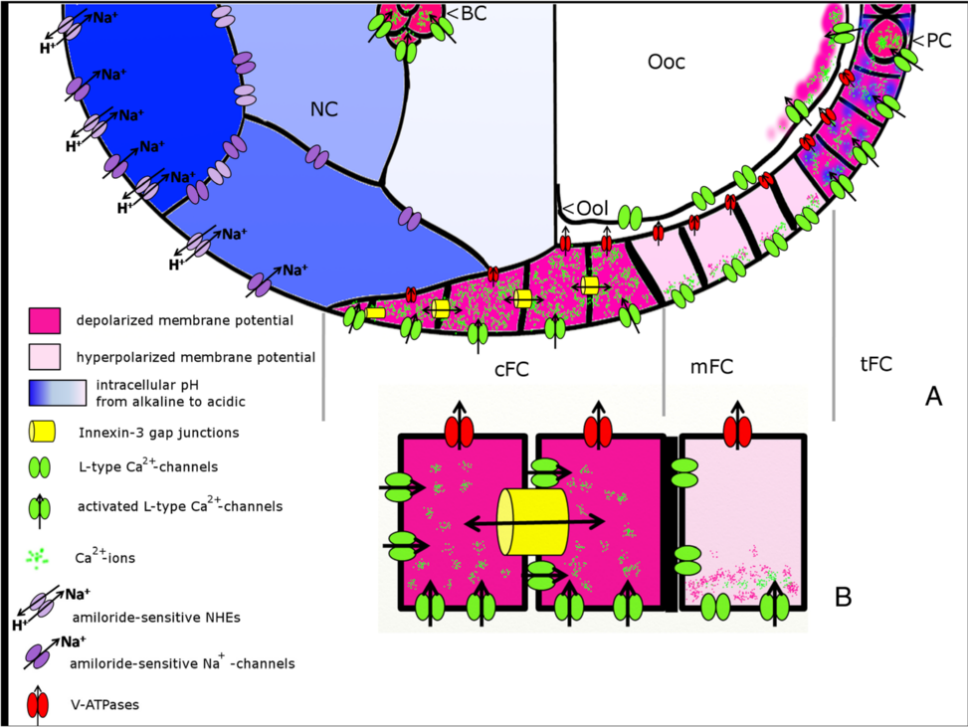
**Summary of V**_**mem**_ **-, pH**_**i**_ **- and ion-transport patterns analysed in the present study.** S9-follicles (for example) are characterized through complex patterns of V_mem_, pH_i_ and distribution or activity of ion-transport mechanisms (for abbreviations, see Figure 1). **A**: Within NC, an anteroposterior pH_i_-gradient, the anterior NC being more alkaline (dark blue) and the posterior NC being more acidic (light blue), was observed. A corresponding pattern was found for amiloride-sensitive Na^+^-transporters. Presumably, the pH_i_-grandient is either generated by asymmetrically distributed NHE, with higher concentrations in anterior NC, or the pH_i_-gradient influences the activity of amiloride-sensitive Na^+^-channels and NHE. The FC-epithelium is subdivided into cFC, mFC, tFC, PC and BC (the sFC are not shown). L-type Ca^2+^-channels as well as V-ATPases are equally distributed in the membranes of cFC, mFC and tFC. V-ATPase concentration is higher on the ventral side. Depolarized regions (cFC, tFC, PC and BC) are characterized through higher Ca^2+^-channel activity. Since inwardly directed Ca^2+^-currents through L-type Ca^2+^-channels depend on V_mem_, we propose a mechanism by which alterations of V_mem_ can be translated into cellular responses through voltage-dependent Ca^2+^-currents. Within each mFC, we found an apicobasal potential gradient (the basal side being more depolarized), as well as an apicobasal gradient of Ca^2+^-channel activity. Innexin-3-containing gap junctions, which are enriched in lateral membranes of cFC, are supposed to regulate V_mem_-, pH_i_- and ion distributions within cFC and to establish a communication border to the neighboring mFC. This border between cFC and mFC, the apicobasal V_mem_-gradient within mFC, the specialized communication via innexin-3-containing gap junctions, and the supposed voltage-gated Ca^2+^-signaling are summarized in more detail in **B**. Despite a growing number of new results, there still exist various missing links between the bioelectric patterns [5-7], the ion-transport patterns [8,9,26,41,65-67] and the distributions of gap-junction proteins [12,46,50] described previously in *Drosophila* ovarian follicles.

Spatial patterns of V_mem_ and pH_i_ related to non-uniform distribution and activity patterns of membrane channels (for summary, see Figure 8) are supposed to generate bioelectric signals during several important steps of oogenesis, e.g. for the regulation of spatial coordinates, migration processes, or reorganization of the cytoskeleton. In recent years, the complex interrelations as well as the physiological and cellular functions of bioelectric phenomena have become increasingly challenging [70]. Analysing the distribution and activity patterns of further ion-transport mechanisms - also in relevant *Drosophila* mutants e.g. [53,71,72] - as well as specific manipulations of V_mem_ and pH_i_ by using appropriate inhibitors will be promising ways to help cracking the bioelectric code [73].

## Abbreviations

BC: Border cells
BSA: Bovine serum albumine
cFC: Centripetal follicle cells
CFDA: 5-carboxyfluorescein diacetate, acetoxymethyl ester
DAPI: 4′,6-diamidino-2-phenylindole
DiBAC: Bis-(1,3-dibutylbarbituric acid) trimethine oxonol
DIC: Differential interference contrast
DMSO: Dimethyl sulfoxide
FC: Follicle cells
Inx3: Innexin 3
mFC: Mainbody follicle cells
NC: Nurse cells
NHE: Na^+^,H^+^-exchangers
Ooc: Oocyte
Ool: Oolemma
PBS: Phosphate buffered saline
PC: Posterior polar cells
pH_i_: Intracellular pH
S: Stage
sFC: stretched follicle cells
SIM: structured-illumination microscopy
tFC: terminal follicle cells
V_mem_: Membrane potential
WFM: Wide-field microscopy
